# Longer sleep duration may negatively affect renal function

**DOI:** 10.1007/s11255-020-02624-6

**Published:** 2020-09-24

**Authors:** Mohsen Mazidi, Niloofar Shekoohi, Niki Katsiki, Maciej Banach

**Affiliations:** 1 Department of Twin Research and Genetic Epidemiology, King’s College London, St Thomas’ Campus, Lambeth Palace Road, London, SE1 7EH UK; 2 Department of Cellular and Molecular Nutrition, School of Nutritional Sciences and Dietetics, University of Medical Sciences, Tehran, Iran; 3 First Department of Internal Medicine, Center for Diabetes, Metabolism and Endocrinology, AHEPA University Hospital, Thessaloniki, Greece; 4 Department of Hypertension, Chair of Nephrology and Hypertension, WAM University Hospital, Medical University of Lodz, Zeromskiego 113, 90-549 Lodz, Poland; 5 Polish Mother’s Memorial Hospital Research Institute (PMMHRI), Lodz, Poland; 6 Cardiovascular Research Centre, University of Zielona Gora, Zielona Gora, Poland

**Keywords:** Mendelian randomization, Sleep duration, Chronic kidney disease, Estimated glomerular filtration rate

## Abstract

**Background:**

Observational studies evaluating the link between sleep duration and kidney function reported controversial results. In the present study, Mendelian randomization analysis was applied to obtain unconfounded estimates of the casual association of genetically determined sleep duration with estimated glomerular filtration rate and the risk of chronic kidney disease.

**Methods:**

Data from the largest genome-wide association studies on self-reported and accelerometer-derived sleep duration, estimated glomerular filtration rate and chronic kidney disease were analysed in total, as well as separately in diabetic and non-diabetic individuals. Inverse variance weighted (IVW) method, weighted median-based method, MR-Egger and MR-Pleiotropy RESidual Sum and Outlier (MR-PRESSO) were applied, as well as the leave-one-out method to rule out the impact of single single-nucleotide polymorphism.

**Results:**

Individuals with genetically longer self-reported sleep duration had a higher chronic kidney disease risk (IVW: *β* = 0.358, *p* = 0.047). Furthermore, in non-diabetics, longer self-reported sleep duration was negatively associated with estimated glomerular filtration rate (IVW: *β* = − 0.024, *p* = 0.020). Similarly, accelerometer-derived sleep duration was negatively related to estimated glomerular filtration rate in the total population (IVW: *β* = − 0.019, *p* = 0.047) and then on-diabetic individuals. No significant association was found between self-reported sleep duration and estimated glomerular filtration rate in the whole population and type-2 diabetes mellitus patients. None of the estimated associations was subjected to a significant level of heterogeneity. MR-PRESSO analysis did not show any chance of outliers for all estimates. The pleiotropy test also indicated low chance of pleiotropy. The leave-one-out method demonstrated that the links were not driven by single-nucleotide polymorphisms.

**Conclusions:**

For the first time, the present study shed a light on the potential harmful effects of longer sleep duration (measured both objectively and subjectively) on kidney function. This finding was observed in the total population and in non-diabetic individuals, but not in those with diabetes. Further research is needed to elucidate the links between sleep duration, estimated glomerular filtration rate and the risk of chronic kidney disease.

## Introduction

Adequate sleep is crucial for the regulation of body metabolism and various physiological functions [1]. Observational studies have previously reported that sleeping patterns are closely linked to morbidity and mortality [2, 3], as well as with chronic metabolic disorders, including type 2 diabetes mellitus (T2DM), respiratory diseases, hypertension and obesity [4–8]. Considering the harmful effects of short sleep duration on glucose and insulin homeostasis, it has been suggested that sleep duration may also influence renal function [9–15]. Furthermore, animal studies showed that the majority of kidney physiological mechanisms (such as the regulation of the renin–angiotensin system, sodium reabsorption, renal blood flow, glomerular filtration, and filtration fraction) are related to the diurnal rhythm [16].

Although the effects of sleep duration on several diseases have been studied, its association with chronic kidney disease (CKD) has not been widely evaluated and the available results are still controversial [9–15]. In this context, a higher risk of CKD has been reported mainly for individuals with shorter sleep duration [9]. In a prospective cohort of 4238 participants from the Nurse Health Study over an 11-year period, shorter sleep duration was associated with a rapid decline in estimated glomerular filtration rate (eGFR) [11]. Moreover, in another prospective study of 6834 Japanese individuals, short sleep duration (≤ 6 h per night) was a predictor of proteinuria [12]. On the other hand, a population-based study of Malay and Indian adults with diabetes showed that long sleep duration (> 8 h per night) was linked to lower eGFR [13]. In the same study, both long (> 8 h) and very short (< 5 h) duration of sleep were associated with albuminuria [13]. Of note, a previous meta-analysis found a direct association of short sleep duration with proteinuria (but not with the risk of CKD) [10]. In another study, a significant association between short sleep duration and higher risk for diabetic nephropathy has been reported [15]. Ohkuma et al. reported that both short and long duration of sleep were related to a higher urinary albumin-to-creatinine ratio (UACR) and albuminuria, whereas long sleep duration was associated with a lower eGFR [14]. However, it should be noted that epidemiological studies provide poor estimates of the role of exposure (sleep duration) on disease development, and thus these studies are prone to bias.

In the present study, we performed Mendelian randomization (MR) [17] analysis to circumvent the limitations of the epidemiological studies (i.e. residual bias, confounding factors and reverse causation), aiming at assessing the associations between sleep duration, eGFR and the risk of CKD. We defined sleep duration by both subjective (i.e. self-reported) and objective (derived by accelerometer) measurements [18].

## Methods

### Study design

A two-sample MR study design was used. Summary statistics were obtained from the largest genome wide association studies (GWAS) on sleep duration and interested outcomes. We applied methods to estimate the unbiased effect of sleep duration on eGFR and the risk of CKD (defined as eGFR < 60 ml/min/1.73 m^2^).

### Genetic instruments for sleep duration

Genotyping, quality control, and imputation procedures in the UK Biobank (UKB) are described elsewhere [19]. From the largest GWAS, 78 SNPs were identified to be associated with sleep duration (self-reported) among individuals of European ancestry (*n* = 446,118) (Table 1). More information can be found elsewhere [20].

**Table 1:** Summary results of the genetic loci of sleep duration obtained in varied methods

SNP	GX	GX SE	EA	OA	EAF
Self-reported					
rs915416	0.019259	0.002495	C	G	0.289947
rs269054	− 0.01364	0.002293	T	A	0.577924
rs12567114	− 0.01483	0.00254	G	A	0.724198
rs62120041	0.026111	0.004575	T	C	0.933902
rs374153	0.017612	0.003103	C	T	0.158085
rs75539574	− 0.03625	0.004065	A	C	0.914208
rs7556815	− 0.04072	0.00274	G	A	0.780856
rs12611523	0.012635	0.002276	A	G	0.545244
rs4538155	− 0.01298	0.002374	C	T	0.352574
rs10173260	− 0.01284	0.002313	T	C	0.393765
rs112230981	0.031528	0.005228	A	G	0.94984
rs17732997	0.012935	0.002288	C	G	0.569098
rs7644809	0.013062	0.002301	T	C	0.421606
rs13088093	− 0.01627	0.002402	T	G	0.663683
rs2192528	0.013369	0.002269	A	G	0.480065
rs17427571	0.013826	0.002435	A	G	0.684313
rs35531607	− 0.01284	0.002273	T	C	0.525917
rs13109404	0.031204	0.004408	T	G	0.928024
rs365663	0.014629	0.002279	A	G	0.545963
rs56372231	− 0.01694	0.0024	C	T	0.665907
rs180769	0.012724	0.002294	T	C	0.424698
rs151014368	− 0.01609	0.00282	G	A	0.793742
rs34556183	0.016923	0.002523	A	G	0.719606
rs80193650	− 0.01684	0.003067	A	G	0.837534
rs9382445	0.014536	0.002334	T	C	0.62305
rs2231265	− 0.01496	0.002699	A	G	0.227711
rs34731055	− 0.01946	0.002948	C	T	0.81911
rs2079070	0.017548	0.002566	C	G	0.264613
rs7806045	0.014792	0.002626	T	C	0.754703
rs330088	− 0.01447	0.002277	T	C	0.452988
rs10973207	− 0.02043	0.003124	G	T	0.842323
rs1776776	0.019963	0.003411	T	C	0.873832
rs12246842	0.013395	0.002274	A	G	0.459815
rs10761674	0.012333	0.002266	C	T	0.477334
rs11190970	0.015379	0.002823	G	A	0.798661
rs7915425	0.019064	0.00299	T	C	0.174682
rs1517572	− 0.01464	0.002295	A	C	0.419464
rs4592416	− 0.01468	0.00227	A	G	0.535593
rs174560	− 0.01358	0.002437	T	C	0.685785
rs12791153	− 0.02355	0.004217	A	T	0.918911
rs1939455	0.020425	0.003561	G	T	0.879446
rs1263056	0.012799	0.002277	A	G	0.519099
rs34354917	0.013746	0.002501	C	A	0.710472
rs11614986	0.016379	0.002951	A	G	0.820952
rs6575005	0.015564	0.002642	T	C	0.757854
rs61985058	− 0.01859	0.003229	C	T	0.856824
rs11621908	0.024095	0.004163	C	T	0.917141
rs8038326	0.01592	0.002541	A	G	0.72691
rs3095508	0.015352	0.002304	C	A	0.593529
rs11643715	− 0.0139	0.002497	C	G	0.709058
rs9940646	0.016946	0.002291	C	G	0.577569
rs7503199	0.014745	0.002564	C	T	0.734267
rs1991556	0.016566	0.002724	G	A	0.773765
rs12607679	0.020139	0.002593	T	C	0.737717
rs10421649	− 0.0133	0.002295	T	A	0.44303
rs2072727	0.013243	0.002285	T	C	0.43617
Accelerometer derived					
rs2660302	0.041	0.006	A	T	0.811
rs113851554	0.11	0.011	G	T	0.943
rs62158170	0.054	0.006	G	A	0.217
rs17400325	0.066	0.012	T	C	0.958
rs72828540	0.041	0.005	T	C	0.752
rs9369062	0.033	0.005	C	A	0.292
rs2975734	0.027	0.005	C	G	0.561
rs13282541	0.032	0.005	C	T	0.739
rs2880370	0.028	0.005	A	T	0.67
rs800165	0.028	0.005	T	T	0.343
rs10138240	0.029	0.005	G	G	0.514

*EA* effect allele, *OA* other allele, *EAF* effect allele frequency, *GX* the per-allele effect on standard deviation units of the telomere length, *GX SE* standard error of GX

We applied on the GWAS performed in the UK Biobank for the accelerometer-driven sleep duration data (an hour increase of nocturnal sleep duration) to be compared with causal estimates obtained using genetic variants associated with self-reported sleep duration. Further details on this process can be found elsewhere [21]. If a single-nucleotide polymorphism (SNP) was unavailable for the outcome GWAS summary statistics, we identified proxy SNPs with a minimum linkage disequilibrium (LD) *r*^2^ = 0.8. To minimize bias in effect estimates induced by correlation between SNPs, we restricted our genetic instrument to independent SNPs not in linkage disequilibrium (*p* = 0.0001). We refer to a set of SNPs that proxy sleep duration as “genetic instruments.”

### Association of genetic instruments with outcome

Genetic associations with renal function were obtained from the largest available extensively genotyped study based on a meta-analysis (*n* = 133,413 individuals with replication in up to 42,166 individuals) [22]. eGFR was estimated using the four-variable modification of diet in renal disease (MDRD) Study equation [22]. CKD was defined as eGFR < 60 ml/min/1.73 m^2^. T2DM was defined as fasting glucose ≥ 126 mg/dl, antidiabetic drug treatment or by self-reports. Kidney function and T2DM were assessed simultaneously.

For GWAS analysis, a centralized analysis plan was applied with each study regressing sex- and age-adjusted residuals of the logarithm of eGFR on SNP dosage levels. Furthermore, logistic regression of CKD was performed on SNP dosage levels adjusting for sex and age. For all traits, adjustment for appropriate study-specific features, such as study site and genetic principal components, was included in the regression and family-based studies appropriately accounted for relatedness.

### MR analysis

We combined the effect of five instruments using inverse variance weighted (IVW) method as implemented in the two sample MR package running under R [version 3.4.2 R Core Team (2017)]. We assessed the heterogeneity using *Q* value for IVW. To address potential effect of pleiotropic variants on the final effect estimate, we performed a sensitivity analysis including weighted median (WM) and MR-Egger. Sensitivity analysis was conducted using the leave-one-out method to identify instruments that might drive the MR results. The WM estimate provides correct estimates as long as SNPs accounting for ≥ 50% of the weight are valid instruments. Inverse variance is used to weight the variants and bootstrapping is applied to estimate the confidence intervals (CIs) [23]. MR-Egger has an ability to make estimates even under the assumption that all SNPs are invalid instruments, as long as the assumption of instrument strength independent of direct effect (InSIDE) is satisfied [23]. However, the InSIDE assumption cannot be easily verified. Average directional pleiotropy across genetic variants was assessed from the p value of the intercept term from MR-Egger [23]. Causal estimates in MR-Egger are less precise than those obtained by IVW MR [24]. Analysis using MR-Egger has a lower false-positive rate but a higher false-negative rate than IVW, i.e. it has a lower statistical power [25].

To assess heterogeneity between individual genetic variant estimates, we performed the Q′ heterogeneity statistic [26] and the MR pleiotropy residual sum and outlier (MR-PRESSO) test [26]. The Q′ statistic uses modified second-order weights that are a derivation of a Taylor series expansion and consider the uncertainty in both numerator and denominator of the instrumental variable ratio [26]. The MR-PRESSO framework detects effect estimates that are outliers and removes them from the analysis. This is done by regressing the variant-outcome associations on variant-exposure associations. A global heterogeneity test is then implemented, comparing the observed distance between residual sums of squares of all variants to the regression line with the distance expected under the null hypothesis of no pleiotropy [27]. Furthermore, we applied on MR-Robust Adjusted Profile Score (RAPS); this method is able to correct for pleiotropy using robust adjusted profile scores. RAPS can provide an unbiased causal estimate in the presence of weak instruments. We consider as results causal estimates that agreed in direction and magnitude across MR methods, passed nominal significance in IVW MR and did not show any evidence of bias from horizontal pleiotropy using heterogeneity tests. To assess the instrumental variable analysis “exclusion-restriction” assumption, we used Ensembl release (https://useast.ensembl.org/index.html) that contains a base of SNP phenotypes.

### Ethics

This investigation uses published or publicly available summary data with no further involvement of participants. No original data were collected for this study. Ethical approval for each of the studies included in the analyses can be found in the original publications (including informed consent from each participant). The study conforms to the ethical guidelines of the 1975 Declaration of Helsinki.

## Results

### Mendelian randomization (MR) analysis

The list of all instruments’ associations for sleep duration (subjective and objective) is shown in Table 1. The results, expressed as *β*-coefficient for sleep duration per 1 standard deviation (SD) increase in outcomes, are presented in Table 2 (for self-reported sleep duration) and Table 3 (for accelerometer-derived sleep duration). With regard to the self-reported sleep duration, individuals with longer sleep duration had a greater risk of CKD (IVW: *β* = 0.358, *p* = 0.047) in the total population. A significant link between longer self-reported sleep duration and lower eGFR was also observed but only among non-diabetics (IVW: *β* = − 0.024, *p* = 0.020). No significant association was found between self-reported sleep duration and eGFR in the whole population (IVW: *β* = − 0.019, *p* = 0.072) and T2DM patients (IVW: *β* = 0.028, *p* = 0.484).

**Table 2:** Results of the Mendelian randomization (MR) analysis for all exposures (for self-reported sleep duration)

Exposures	MR				Heterogeneity			Pleiotropy		
	Method	Beta	SE	*p*	Method	*Q*	*p* value	Intercept	SE	*p*
CKD	MR-Egger	0.9034	0.6866	0.1966	MR-Egger	45.34	0.1368	− 0.0093	0.011	0.415
	WM	0.4764	0.2685	0.07603						
	IVW	0.3583	0.1856	0.047	IVW	46.2	0.1428			
	RAPS	0.4413	0.1941	0.02299						
eGFR										
Totali	MR-Egger	− 0.0409	0.03856	0.2959	MR-Egger	45.05	0.1434	0.00038	0.00064	0.562
	WM	− 0.01982	0.01604	0.2165						
	IVW	− 0.01913	0.01066	0.0727	IVW	45.48	0.1597			
	RAPS	− 0.02149	0.01091	0.04876						
Non-DM	MR-Egger	− 0.06524	0.04006	0.1121	MR-Egger	42.11	0.2235	0.00069	0.00066	0.302
	WM	− 0.03157	0.01585	0.04633						
	IVW	− 0.02476	0.01066	0.02016	IVW	43.39	0.2175			
	RAPS	− 0.02577	0.01084	0.01744						
DM	MR-Egger	0.04857	0.1499	0.7478	MR-Egger	37.96	0.3801	− 0.00035	0.0025	0.888
	WM	0.02369	0.06189	0.7019						
	IVW	0.02804	0.04007	0.4841	IVW	37.98	0.4245			
	RAPS	0.02957	0.04148	0.4759						

*WM* weighted median, *IVW* inverse variance weighted, *SE* standard error, *beta* beta-coefficients, *MR* Mendelian randomization, *CKD* chronic kidney disease, *eGFR* estimated glomerular filtration rate, *T2DM* type 2 diabetes mellitus

**Table 3:** Results of the mendelian randomization (MR) analysis for all exposures (for accelerometer derived sleep duration)

Exposures	MR				Heterogeneity			Pleiotropy		
	Method	Beta	SE	*p*	Method	*Q*	*p* value	Intercept	SE	*p*
CKD	MR-Egger	1.557	0.7086	0.07938	MR-Egger	2.765	0.7362	− 0.066	0.028	0.0665
	WM	0.1731	0.2271	0.4459						
	IVW	− 0.04924	0.204	0.8093	IVW	8.232	0.2216			
	RAPS	− 0.01708	0.2092	0.9349						
eGFR										
Total	MR-Egger	− 0.03524	0.04022	0.421	MR-Egger	1.4	0.9243	0.00065	0.0016	0.706
	WM	− 0.01553	0.01165	0.1824						
	IVW	− 0.01969	0.01	0.04702	IVW	1.559	0.9555			
	RAPS	− 0.01978	0.01038	0.05679						
Non-DM	MR-Egger	− 0.03565	0.04178	0.4325	MR-Egger	2.015	0.847	0.00043	0.0017	0.808
	WM	− 0.02424	0.01263	0.055						
	IVW	− 0.02527	0.01038	0.01486	IVW	2.081	0.9121			
	RAPS	− 0.02541	0.01082	0.01891						
DM	MR Egger	− 0.2975	0.1684	0.1377	MR-Egger	4.758	0.4461	0.011	0.0068	0.153
	WM	− 0.01371	0.05743	0.8113						
	IVW	− 0.02287	0.04688	0.6256	IVW	7.589	0.2698			
	RAPS	− 0.02561	0.04763	0.5908						

*WM* weighted median, *IVW* inverse variance weighted, *SE* standard error, *beta* beta-coefficients, *MR* mendelian randomization, *CKD* chronic kidney disease, *eGFR* estimated glomerular filtration rate, *DM* diabetes

With regard to the accelerometer-derived sleep duration, individuals with longer sleep duration had a lower eGFR both in the total population (IVW: *β* = − 0.019, *p* = 0.047) and the non-diabetics (IVW: *β* = − 0.025, *p* = 0.014), but not among T2DM patients (IVW: *β* = − 0.022, *p* = 0.625). Furthermore, there was no significant link between sleep duration and the risk for CKD in the total population, the non-diabetics and the T2DM group.

None of the IWV estimates showed heterogeneity. MR-PRESSO analysis did not show any possibility of outlier for all of the estimates. The pleiotropy test, with very negligible intercept and insignificant p value, also indicated low chance of the pleiotropy for all of our estimations (all *p* > 0.539). The results of the MR-RAPS were identical with the IVW estimates, highlighting again a low likelihood of pleiotropy. The results of the leave-one-out method demonstrated that the links were not driven by single SNPs.

## Discussion

In the present study, we performed a MR analysis to evaluate the impact of sleep durationon the risk of CKD and renal function indexes in a casual mode. Potential harmful effects of longer sleep duration (measured both objectively and subjectively) on kidney function were found. This finding was observed in the total population and in non-diabetic individuals, but not in those with T2DM, with low level of the heterogeneity and chance of pleiotropic.

There are several plausible mechanisms regarding the association of sleep duration with renal function that need to be further elucidated. Most renal physiological processes follow a circadian rhythm, including the regulation of sodium excretion, renin–angiotensin system and blood pressure [28]. Therefore, disruption in sleeping patterns (short or long sleep duration) may negatively affect this chronobiological process and lead to renal dysfunction. In this context, disruption in cyclic behavioural patterns in animal models was associated with proteinuria, glomerulosclerosis, tubular hyperplasia, and renal fibrosis [29]. Furthermore, sleeping 5 h or fewer per night was more related to the development of hypertension and T2DM during a 10-year follow-up period compared with sleeping 7 h per night [5, 30]. In short term studies, lasting 3–6 weeks, sleep duration was a modifiable predictor of insulin resistance and blood pressure [31, 32]. Short sleep duration was associated with a rapid decline in renal function independently of established CKD risk factors such as blood pressure and insulin resistance [11]. Moreover, sleep duration may affect the levels of pro-inflammatory markers such as interleukin-6 and C-reactive protein [33, 34] that play an important role in CKD pathogenesis [35]. Obstructive sleep apnea (OSA) has been linked to the risk of CKD [36, 37] via enhanced sympathetic activation, oxidative and nitrosative stress, and impaired microvascular and endothelial function in T2DM patients, leading to renal dysfunction [38, 39]. Taking into consideration the association between sleep duration and OSA [40], sleep duration may also affect the risk for CKD development via this pathway.

A few studies investigated the relationship between sleep duration and CKD with controversial results. Cross-sectional studies have previously reported that advanced CKD can lead to a higher prevalence of several sleep disorders, including reversal of day–night sleep patterns, increased sleep latency, and fragmented sleep related to sleep apnea or restless leg syndrome [9]. In this context, shorter sleep duration (i.e. < 5 h) measured by actigraphy was significantly more prevalent in non-dialysis dependent CKD and end-stage renal disease (ESRD) patients compared with individuals with normal kidney function [41]. Ohkuma et al*.* [14] reported that both short and long sleep duration were associated with higher UACR (urinary albumin-to-creatinine ratio) and albuminuria. Moreover, consistent with our observations, lower eGFR correlated with long sleep duration independently of potential confounders [14]. In a population-based cross-sectional study of 1258 T2DM patients (aged 40–80 years), long sleep duration (> 8 h) was associated with renal insufficiency compared with normal sleep duration (7–8 h) [13]. In the same study, both long and very short duration of sleep (< 5 h) were linked with albuminuria; after adjusting for potential confounders, only long sleep duration remained significantly related to 2.3-fold higher odds of low eGFR [13]. Another study of 6834 Japanese adults (aged 20–65 years) reported that proteinuria was more frequently developed in individuals who slept 6 or fewer hours than those who slept for 7–8 h per night; however, eGFR did not differ between these individuals [12]. Similarly, sleep parameters were not associated with a 5-year change in eGFR in a prospective study of 463 adults (aged 32–51 years) [42]. However, each 1 h decrease in sleep duration was significantly related to a 1.5 ml/min/1.73 m^2^ higher eGFR [42].

In contrast to our results, a systematic review and meta-analysis of 6 observational studies with 252,075 individuals and 3 observational studies with 37,197 participants reported a positive significant association between short sleep duration and proteinuria, but no association between short sleep duration and CKD [10]. In another study, short (but not long) sleep duration was related to diabetic CKD (DKD), defined as the presence of albuminuria and eGFR < 60 ml/min/1.73 m^2^ [15]. Furthermore, a prospective cohort study of 4238 individuals from the Nurse Health Study over an 11-year period reported that shorter sleep duration was associated with a rapid reduction in eGFR [11]. Of note, women who slept 5 h or less per night experienced the quickest decline in renal function, whereas the slowest rate of decline was observed in those who reported sleeping 7–8 h per night [11]. Moreover, albuminuria was twice as prevalent among those who slept 5 or fewer hours per night compared with those who reported 7–8 h of sleep per night [11]. However, some limitations should be considered for this study. First of all, sleep duration was collected 3 years prior to initial eGFR measurement. Second, the U-shaped association between sleep duration and rapid decline in renal function was not assessed due to the limited number of individuals reporting sleep duration of 9 h or more [11]. Third, the study population was limited to women, and most individuals were white. Therefore, findings cannot be applied to men and other racial groups.

The present study has several strengths, e.g. the possibility of reverse causation was minimized, genotypes were assumed to be randomly distributed with respect to confounders, the ability to perform an array of sensitivity analyses was enhanced by the use of pleiotropy-robust methods. There are also certain limitations, including the inability to thoroughly evaluate individual-level confounding factors and horizontal pleiotropy.

In conclusion, potential harmful effects of longer sleep duration (measured both objectively and subjectively) on kidney function was observed in a casual mode in the present MR analysis. This finding was observed both in total population and non-diabetic individuals, but not in those with T2DM. Further research is needed in this field to elucidate the associations between sleep duration and renal function.
